# Alternative reproductive tactics are associated with sperm performance in invasive round goby from two different salinity environments

**DOI:** 10.1002/ece3.6657

**Published:** 2020-09-06

**Authors:** Leon Green, Jan Niemax, Jens‐Peter Herrmann, Axel Temming, Charlotta Kvarnemo

**Affiliations:** ^1^ Department of Biological and Environmental Sciences University of Gothenburg Gothenburg Sweden; ^2^ Linnaeus Centre for Evolutionary Marine Biology University of Gothenburg Gothenburg Sweden; ^3^ Department of Biology Institute of Marine Ecosystem and Fishery Science University of Hamburg Hamburg Germany

## Abstract

During male–male competition, evolution can favor alternative reproductive tactics. This often results in a dominant morph that holds a resource, such as a nest for egg laying, which competes with a smaller sneaker morph that reproduces by stealing fertilizations. The salinity environment can influence male growth rates, for example, via osmoregulatory costs, which in turn may influence the use of sneaker tactics for small males competing for mating opportunities. Salinity can also affect sperm directly; however, little is known of how salinity influences sneaker tactics through sperm performance. We sampled males of the invasive round goby (*Neogobius melanostomus*) from two environments, a freshwater river and a brackish estuary. This fish has two male morphs: nest‐holding dark males and non‐nest‐holding light males. We examined the role of water salinity of 0, 8, and 16 on sperm performance and found that for estuarine males, a salinity of 0 reduced sperm velocity compared to a salinity of 8 and 16. Riverine males had low velocity in all salinities. Sperm viability also decreased by over 30% in 0 salinity, compared to 8 and 16, for fish from both environments. Gobies produce ejaculate contents in specialized glands that could in theory shield sperm in an adverse environment. However, gland contents did not improve sperm performance in our tests. Body mass and age estimates indicate that riverine males invested more in somatic growth compared to estuarine males. Estuarine light morph males had a high enough gonadosomatic index to indicate sneaker tactics. We propose that when sperm performance is low, such as for the riverine males, sneaker tactics are ineffective and will be selected against or phenotypically suppressed. Instead, we interpret the increased investment in somatic growth found in riverine males as a life‐history decision that is advantageous when defending a nest in the next reproductive season.

## Graphical abstract

For a male fish too small to defend mating resources such as a female or her eggs, siring offspring can be achieved by sneaking into another male's nest and ejaculating there. In the invasive round goby, this alternative tactic requires sperm to function long enough to fertilize the eggs. This fish has spread into a range of salinities, but for some populations, fresh water decreases sperm velocity and lifespan, and severely limits the use of these alternative reproductive tactics to the point where they may disappear from use.

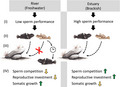

## 1 INTRODUCTION

During competition for fertilization opportunities, males are expected to employ reproductive strategies that maximize their lifetime reproductive success (Gross, 1996; Parker, 1990a; Parker, 1990b; Taborsky, Oliveira, & Brockmann, 2008). Some species have evolved alternative reproductive tactics, commonly expressed as two phenotypic male morphs, a “parental” male morph (sometimes called “bourgeois,” conventional or type I) that is comparatively large, resource‐holding, dominant and aggressive, and a “sneaker” morph (parasitic male or type II), which is smaller and specializes in parasitic spawnings (Taborsky, 1998). The latter is usually done by parasitizing the dominant males' reproductive attempts by mimicking females or by acting subordinate and insignificant (Barlow, 1967; Dominey, 1980; Gonçalves, Almada, Oliveira, & Santos, 1996). However, parasitic spawning can also occur among “bourgeois” males (Petersen & Warner, 1998; Singer, Kvarnemo, Lindström, & Svensson, 2006; Taborsky, 1998). These reproductive tactics are known all across the animal kingdom, from arthropods (Brockmann & Penn, 1992) to molluscs (Norman, Finn, & Tregenza, 1999) to vertebrates (Taborsky, 2008).

Energy allocation decisions in both reproductive tactics can involve trade‐offs between different behavioral and physiological efforts, including investment in gonads, reproductive fluids, and seminal vesicles (Snook, 2005). Males using sneaker tactics reproduce under sperm competition, but can avoid the cost of courtship and parental care, behaviors that are commonly expressed in “bourgeois” males (Taborsky et al., 2008). By evolving an ability to switch tactics during their lifetime, individuals can be better suited to employ the most economic trade‐off available (Parker & Pizzari, 2010; Taborsky, 1998; Tomkins & Hazel, 2007). How the development or switch into a specific reproductive morph occurs varies between species and conditions. Cues from the natural (Farr, Travis, & Trexler, 1986; Hutchings & Myers, 1994; Sigurjónsdóttir & Gunnarsson, 1989) and social environment (Kodric‐Brown, 1986; Takegaki, Svensson, & Kvarnemo, 2012), as well as the males' own physiological condition (Immler, Mazzoldi, & Rasotto, 2004; Magnhagen, 1992), are often involved. The environmental influence has commonly been studied through effects on the males' own physiological condition, for example, through size (Bleeker, De Jong, Van Kessel, Hinde, & Nagelkerke, 2017; De Fraipont, FitzGerald, & Guderley, 1993; Flemming, 1996; Gross, 1984; Magnhagen, 1992), as a small male is better off using sneaking tactics rather than trying to monopolize access to females (Gross, 1991b). However, different reproductive tactics can also have sperm that are specialized to function better under certain chemical or physiological conditions (Locatello, Poli, & Rasotto, 2013; Nakanishi & Takegaki, 2019). In theory, sperm from different tactics could be more or less successful in different environments, which could affect the expression of the tactic itself.

In fishes, changes in salinity can trigger sperm activation (Alavi & Cosson, 2006), but also deplete them of energy for movement and cellular function (Cosson et al., 2008). Because of this, sperm are sensitive to salinity and most fishes have sperm that are adapted to function only in their local salinity (Alavi & Cosson, 2006; Browne et al., 2015; Morisawa, 2008). For fish species that occupy both fresh and salt water during their lives, the common solution is to reproduce in only one environment, anadromously or catadromously (McDowall, 1997). Changing between fresh water and salt water is associated with major physiological change in fishes (Kultz, 2015). Salmonids are textbook examples of these phenotypic alterations, reorganizing osmoregulation, color, and feeding habits (Groot, Margolis, & Clarke, 2002). Migration is also known to be related to the choice of reproductive tactics used, through the condition state of the male: In the Atlantic salmon, small, nonmigrating freshwater males employ sneaker tactics during the reproductive period (Flemming, 1996; Gross, 1991b). However, other fish taxa that live in a wide range of salinities have evolved adaptations that allow them to reproduce in many different salinities, due to a generally wide tolerance or plasticity (Behrens, Van Deurs, & Christensen, 2017), or to local adaptation by different subpopulations along their geographic distributions (Green, Havenhand, & Kvarnemo, 2020; Svensson et al., 2017).

So far, none of the species with alternative reproductive tactics that reproduce in a range of different salinity conditions have been studied in terms of how salinity affects their choice of the best tactic to adopt. Gobies (Teleostei: Gobiidae) belong to a family of fishes where many species show condition‐dependent reproductive tactics (Patzner, Van Tassel, Kovacic, & Kapoor, 2011). Many of them are euryhaline (Fig. 4 in Adrian‐Kalchhauser et al., 2017). Several of them are also invasive (Wonham, Carlton, Ruiz, & Smith, 2000) and have established themselves across a range of salinities (Behrens et al., 2017; Green et al., 2020). One of the most well‐studied of the euryhaline invasive gobies is the round goby (*Neogobius melanostomus*, Pallas 1814). In 1990, the species was inadvertently introduced from the Black Sea region to the Great Lakes in North America and to the Baltic Sea (Kornis, Mercado‐Silva, & Vander Zanden, 2012). It now also occurs in many European rivers (Kornis et al., 2012). The main vector of transport is ships (Kotta, Nurkse, Puntila, & Ojaveer, 2016), and the mechanisms of spread are likely ballast water (Kornis et al., 2012) and egg attachment on boats (Hirsch et al., 2016), possibly also on larger ships. Though localized overwinter migration into deeper waters was observed in 36% of tagged individuals in a survey (Christoffersen, Svendsen, Behrens, Jepsen, & van Deurs, 2019), the round goby does not show long‐distance migratory behavior comparable to the previously mentioned salmonids (Azour et al., 2015; Behrens et al., 2017; Christoffersen et al., 2019). As a consequence, it hatches, reproduces, and dies in the same region, often in the same salinity range. The species has a benthic lifestyle, where they occupy a niche as predators on invertebrate fauna (Oesterwind et al., 2017). Reproduction occurs from April to September (in some regions possibly into November) and larvae hatch from a clutch of eggs with fully developed gut systems and fins, ready to start feeding on plankton from day 1 (Kornis et al., 2012). Age of sexual maturation differs between sexes, with females maturing in their first or second year, and males in their second or third (Kornis et al., 2012). The effect of age on reproductive tactics has not yet been studied in round goby, but size and growth rate in the first year seem to be determining factors (Marentette, Fitzpatrick, Berger, & Balshine, 2009; Somerville et al., 2019). Males express two different reproductive tactics: either guarding a nest and courting females to lay their eggs there, or parasitically spawning in other males' nests (Corkum, MacInnis, & Wicket, 1998; Marentette et al., 2009; Meunier, Yavno, Ahmed, & Corkum, 2009). In round goby, the reproductive tactic can be determined from the males' color morph, since nest‐holding, “bourgeois,” males develop a distinct dark (often jet black) ornamental coloration (Bleeker et al., 2017; Marentette et al., 2009; Meunier et al., 2009; Somerville et al., 2019; Yavno & Corkum, 2010), and can therefore readily be distinguished from males without a nest that are lighter in color (Marentette et al., 2009). These two tactics can be specializations in their own right, but in some other goby species, nest‐holding males are also known to engage in parasitic spawnings (Jones, Walker, Lindström, Kvarnemo, & Avise, 2001; Singer et al., 2006).

Sperm competition is common among gobies and apart from regular ejaculation, nest holders engage in “asynchronous spawning” (Marconato, Rasotto, & Mazzoldi, 1996). This is done by embedding inactive sperm in a slowly dissolving mucus, attached to the nest ceiling before the female lays her eggs there. This allows the male to guard the nest against competitors while the eggs are laid, as his sperm are gradually released from the dissolving mucus and become activated in the water and fertilize the eggs (Marconato et al., 1996; Mazzoldi, Scaggiante, Ambrosin & Rasotto 2000; Scaggiante, Mazzoldi, Petersen, & Rasotto, 1999). This process can take time, and eggs of the grass goby (*Zosterisessor ophiocephalus*, Pallas 1814), a species closely related to round goby (and with similar ecology), can be fertilized 40 hr after laying (Scaggiante et al., 1999). Asynchronous spawning behavior has also been observed in the round goby (Meunier et al., 2009), although the presence of sperm in the mucus has not been investigated.

The mucus is produced in a pair of specialized organs, the sperm duct glands (SDGs), which have been found across the entire goby family tree (Miller, 1984). The nongametic components of the ejaculate, composed in part of SDG contents, in the form of mucus, together with proteins and possibly adenosine triphosphate (ATP), have been shown to energize sperm in several goby species (Green & Kvarnemo, 2019; Locatello et al., 2013; Poli, Locatello, & Rasotto, 2018; Young & Fox, 1937). Nest‐holding goby males are more reliant than sneaker males on SDG contents for their nest preparation and spawning tactics, and consistent with this, nest holders typically have larger SDGs than males without a nest (Immler et al., 2004; Svensson & Kvarnemo, 2007) or sneaker morph males (Kvarnemo, Svensson, & Manson, 2010).

Despite the sensitivity of fish sperm to salinity, the round goby has been reported from salinities ranging from 0 to 40 practical salinity units (PSU) (Kornis et al., 2012). Since SDG contents have been found to improve sperm performance in other goby species (Green & Kvarnemo, 2019; Locatello et al., 2013; Poli et al., 2018), these glands could be an adaptation that facilitates rapid range expansion of the round goby by mitigating detrimental salinity effects.

Two sperm traits commonly studied are sperm velocity and sperm viability. Sperm velocity has been shown to be important for fertilization success, especially during sperm competition in externally fertilizing species (Rudolfsen, Figenschou, Folstad, & Kleven, 2008; Gage et al., 2004). Sperm viability is particularly important in animals with a long time between ejaculation and fertilization, such as honey bees (Collins, Williams & Evans, 2004). In fish with external fertilization, sperm are typically very short‐lived (Alavi & Cosson, 2006), but gobies provide an astonishing exception to this rule (Green & Kvarnemo, 2019). It has been hypothesized that long‐term viability and motility are important traits due to the prolonged spawning session many gobies engage in (Marconato et al., 1996; Nakanishi & Takegaki, 2019).

In this study, we compare round goby males of estuarine and riverine origin and assess how salinity and SDG contents affect sperm velocity and sperm viability. We also investigate how condition and investment in reproductive tissues may differ between males from the two environments and between males of different reproductive tactics (indicated by dark and light color morphs). We sampled fish from a population in a brackish estuary in the southern Baltic Sea (estimated to be 11 years old at the time of sampling) and the river Elbe (estimated to be 9 years old) (Hempel & Thiel, 2013). Since the round goby has its evolutionary history in brackish water, and sperm performance has been reported to decline in lower salinities (Green et al., 2020), we hypothesize that (I) sperm viability is lower in freshwater, but that this effect is ameliorated by ejaculate components from the SDGs. As a consequence of this, we hypothesize that (II) males living in fresh water invest more in SDG content to increase sperm viability. Furthermore, we hypothesize that (III) energy status and body size is lower for gobies in the rivers, due to higher osmoregulatory costs.

## 2 MATERIALS AND METHODS

### 2.1 Study species

Experiments were conducted within the permit nr 59/16 from Amt für Vebraucherschutz, Veterinärwesen und Lebensmittelüberwachung, Hamburg. Round goby (*N. melanostomus*, Pallas 1814) males were caught at an estuarine site close to Travemünde, Bay of Lübeck, Germany (*N* = 20, 53°53′46.5″N 10°47′53.6″E), and a freshwater site, in the River Elbe, Hamburg, Germany (*N* = 15, 53°32′56.9″N 9°59′10.3″E), during several sampling events between April and June 2017. Regional sea surface salinity in the Bay of Lübeck during 2017 varied from 10 to 19 PSU and averaged temperature during sampling period was 14°C (data from the COMBINE Program https://ocean.ices.dk/Helcom/). River Elbe is a freshwater river (0 PSU), and average temperature (measured in 2016 during a separate study) over the sampling period was 16°C. Fish were caught using angling with hook and line, and thawed, peeled shrimp (*Pandalus borealis*) as bait. All animals survived the angling, transport and captivity, until they were sacrificed for the experiment. When caught, the fish were sexed and their readiness to spawn was estimated, based on examination of the shape of their genital papilla (following Marentette et al., 2009). Males estimated to be reproductively active were kept for the experiment (the other fish were released immediately after catch) and transported to the aquarium facilities of the Marine Ecology and Fisheries Science, at Hamburg University, in aerated and insulated boxes within 4 hr of catch. Once there, the fish were kept in plastered fiberglass tanks (tank 140 cm × 140 cm, water depth ~ 50 cm, 22 ± 5 males per tank) connected to a recirculating flow system of their respective natural salinity (0 or 16 PSU) at 16°C, for a maximum of 14 days. During this time, they were fed commercial salmon feed (3 mm pellets, Alltech Coppens, Helmond, The Netherlands) ad libitum, once a day. Although the feeding may have increased their energy status to some degree, all fish were treated the same way, and due to the relatively short time in the laboratory, it was estimated to not have affected their somatic growth or energy status markedly.

### 2.2 Physiological data

When sampling, a male was randomly caught from the holding tank with a hand net, its color morph (dark or light) was visually determined and its reproductive readiness reconfirmed (see above). After this the fish was euthanized by a concussive blow to the head, followed by cutting the afferent gill arteries to stop blood flow to the brain. The total length was measured to the nearest 1 mm using a measuring board. Total body mass was measured to the nearest 1 g using a digital scale (BL3100, Sartorius, Göttingen, Germany). The fish was then dissected and testes and SDGs were weighed separately to the nearest 1 µg using a digital scale (LE26P, Sartorius, Göttingen, Germany). These organs were then used for the sperm velocity and viability essays (see below). After the sperm essays, the liver was dissected from the body and weighed to the nearest 1 µg. Gut and intestine length can in theory differ depending on tactics (nest holders likely feed less), so to control for this, the gut and intestines were eviscerated and the somatic tissue mass was measured without internal organs, weighed to the nearest 1 g. The somatic tissue and liver were then dried for 24 hr using a drying oven (Thermocenter tc 44, SalvisLab, Rotkreuz, Schweiz) at 90°C before being weighed again for dry liver mass and somatic dry mass. To obtain age estimates, one saccular otolith per male was extracted, cleaned, submerged in water, and visually graded (with annuli counted from the core to the edge) at 50× magnification using a microscope (Axio Vert.A1, Carl Zeiss AG) with origin of the fish blinded to the researcher.

### 2.3 Sperm essay preparation

Since we aimed to examine potential effects of SDG contents on sperm velocity and viability, the testis and SDGs were individually dissected from the fish within 1 min of sacrificing it, using stainless steel forceps and scissors (curved, sharp point, 4 in., Sigma‐Aldrich Co, St. Louis, MO, USA). The two testes were then placed into two separate 1.5‐ml microcentrifuge tubes (Eppendorf, Hamburg, Germany), one testis without, and one together with, its SDG. This created two separate ejaculate treatment conditions: sperm only and sperm with SDG contents. The organs were incised 5 times each using scissors (same model as above) and the content diluted with 60 μl calcium‐free Ringer's solution at 16°C (Karila, Jönsson, Jesen, & Holmgren, 1993) for a roughly double increase in liquid volume and to prevent sperm activation (confirmed by visual inspection under microscope at 100× magnification, same model as above). The sample was stirred using a Vortex (Vortex‐Genie 2, Scientific Industries, Bohemia, NY, USA) 3 times for 1 s in rapid succession. The sperm were then activated by transferring 25 μl of each suspension to a new microcentrifuge tube, filled with 750 μl filtered water of 3 different salinities (0 PSU, 8 PSU, and 16 PSU, mixed from artificial sea salt (Instant Ocean, St. Blacksburg, VA, USA) and tap water suitable for drinking) using a micropipette (Transferpette, BrandTech Scientific, Essex, CT, USA). This was done in an effort to mimic the natural way the ejaculate would mix with water in the external environment. This essay created a total of six different treatment combinations: sperm with or without SDG contents, in three different salinities. The six tubes were kept at 16°C in a thermostatic bath during the entire experiment except during vortexing. The tests of these samples were performed identically, and the order was determined by randomly selecting a sample for the measurements described below.

### 2.4 Sperm velocity measurements

Video recordings of sperm velocity were created by transferring 45 μl from the above sample to a 2% (w/v) albumin‐coated glass slide fitted with an O‐ring, and covered with an albumin‐coated coverslip that acted as a lid to suspend the sperm suspension (Havenhand & Schlegel, 2009). This was repeated three times for three technical replicates per male, salinity, and SDG content treatment. The three drops, placed on the same slide, were immediately filmed in rapid succession using a camera (PixeLINK PL‐D725, Pixelink , Ottawa, Canada) fitted to a microscope (same as above) with a 10× magnification objective and standard contrast and illumination, for 15 frames (frame rate: 30 frames/s, size: 2,592 × 2,048 pixels, exposure time: 10 ms, gain: 0, gamma: 0.1). Analysis of the videos was done through the computer‐assisted sperm analysis (CASA) plugin (Wilson‐Leedy & Ingermann, 2007) for ImageJ (National Institutes of Health, Bethesda, MD, USA) following standard procedures (Purchase & Earle, 2012) and CASA settings reported in the supplementary information in Green et al. (2020). The sperm velocity parameter chosen was velocity of the curvilinear path (VCL) to allow for comparison with previous studies (Green et al., 2020; Marentette et al., 2009). Since the procedure was replicated, the velocity measurements from each of the three technical replicates were used to calculate an average for each male and treatment condition. This average sperm velocity was the value used in the analyses.

### 2.5 Sperm viability measurements

Sperm viability was measured 10 min (±1 min) after the sample was created (see above), using the following procedure: The sperm sample was vortexed for 3 × 1 s, and 100 μl was transferred to a separate 600‐μl microcentrifuge tube (Eppendorf). This sample was stained using 1 μl diluted SYBR14 (1 parts SYBR14 to 49 parts DMSO) and 5 μl propidium iodide (diluted 1 parts propidium iodide to 4 parts with DMSO) (LIVE/DEAD® Sperm Viability Kit, L7011, Life Technologies, Thermo Fisher Scientific) (Garner & Johnson, 1995). The sample was then vortexed for 3 × 1 s, and 3 drops of 25 μl of the suspension were transferred to a microscope slide and allowed to spread into separate patches of thin film, to minimize the depth of field and avoid excess movement. The microscope (same as above) was then focused on the glass surface, and two photographs were taken with a camera (same as above), first illuminated with green light (Cy 3 filter, 520–560 nm) to excite the pigments in dead sperm cells, and then blue light (GFP filter, 450–490 nm), to excite the pigments in living sperm cells. The following camera settings were used: size 2,592 × 2,048 pixels, exposure time 500 ms, gain 18.06, gamma 4. This was replicated on each of the three drops, resulting in three technical replicates per male and treatment. The photographs were later filtered digitally and analyzed using ImageJ (same as above) to automatically count the number of dead and alive sperm for each photographed technical replicate. From these counts, sperm viability was calculated as the number of live sperm divided by the total number of sperm. The viability measurements from each technical replicate were used to calculate an average for each male and treatment condition. All calculations were performed identically (i.e., Rep_1_ + Rep_2 _+ Rep_3 _/ 3), regardless of the variance between technical replicates. This average sperm viability was the value used in the analyses.

### 2.6 Statistical analysis

Statistical analyses were done using SPSS, version 25 (IBM, Armonk, NY, USA), and the packages lme4 (Bates, Mächler, Bolker, & Walker, 2015) and lmerTest (Kuznetsova, Brockhoff, & Christensen, 2017) in R version 3.3.3 (R Core Team, 2013).

Salinity and the males' environmental origin were first tested for their effects on sperm velocity and viability, using linear mixed models. Because the sperm of each male were tested in three different salinities, male ID was included as random factor to control for the repeated measurement. Salinity treatment (0, 8, and 16 PSU) and environment (river or estuary) were used as fixed factors. Sperm velocity and sperm viability were used as response variables in two separate models. Models were visually explored to control for randomness in the residuals versus fitted values, similarity of the theoretical and observed quantiles, high influence points, and the frequency distribution of residuals using the “plot(lm)” function inherent in R. The models were analyzed for multicollinearity by assessing variance inflation factors, using the “vif(lm)” from the package car in R. Salinity showed a vif score of above 5 (5.44 for both sperm velocity and viability) but the term was kept in both models based on the expectation of a biological relatedness between salinity and the males environment. Both response variables were also tested for assumptions of normality of residuals and homogeneity of variances. The sperm viability data were non‐normally distributed and did not meet assumptions of sphericity. *p*‐values for fixed effects were generated using Satterthwaite approximations, to reduce the risk of type 1 errors when analyzing small sample size datasets of unbalanced data (Luke, 2017), and tested using type III sums of squares. We followed Hendrix, Carter, and Scott (1982) in that models were sequentially simplified by first removing any nonsignificant interaction terms until a significant effect was found. The random factor (male ID) was consistently kept in the model. Post hoc tests of comparison were performed using Tukey's range tests (within salinities or environments) and Welch's *t* tests (for a priori expectations of differences between groups).

To also test whether high sperm velocity may have resulted in reduced sperm viability, we analyzed viability in each respective group's local salinity (0 PSU for riverine males and 16 PSU for estuarine males). Since no repeated measurements were included for this test, we analyzed the data using a general linear model. Sperm velocity was used as covariate and environment (river or estuary) and color morph (light morph or dark morph) as factors. Model simplification was performed as described above.

We then proceeded to test whether SDG contents or color morph had an effect on sperm velocity and sperm viability when males were spawning in their local salinity, also using linear mixed models. The males from the river and the estuary were tested separately, and only measurements from their local salinity (0 PSU for riverine males and 16 PSU for estuarine males) were used. Because each male's sperm were tested with or without SDG contents, male ID was again included as random factor to control for the repeated measurement. SDG treatment (with or without SDG contents) and color morph (light morph or dark morph) were used as fixed factors. Sperm velocity and sperm viability were used as response variables. Model exploration, tests of assumptions of normality and homogeneity of variances, *p*‐value estimation, and model simplification were performed as described above for the previous mixed models.

All tissue masses were log‐transformed before statistical analyses. Logistic regression was used to investigate what factors best predicted male color morph, using age estimate, total length, and body mass as independent variables in the analysis.

In our analyses of relative size of internal organs (here: testes, SDG, and liver), we used somatic body mass (*i.e.,* without internal organs) to avoid autocorrelations that can arise if body size is measured as total body mass and analyzed together with the mass of internal organs (Tomkins & Simmons, 2002; Stoltz, Neff & Olden, 2005). We analyzed testes mass, SDG mass, and liver dry mass using general linear models, with male origin and male color morph as factors. To control for allometry (i.e., larger individuals having larger internal organs), somatic body mass was used as covariate. Model exploration, tests of assumptions of normality and homogeneity of variances, *p*‐value estimation, and model simplification were performed as described above for the previous mixed models. Since log SDG mass did not show homogeneity of variances, a control model using heteroscedasticity‐consistent standard errors was also calculated and compared with the standard model. As both showed *p*‐values within the same range, the standard model was accepted.

Though criticized (Tomkins & Simmons, 2002), ratio indices are still in use in scientific practice. We therefore also included gonadosomatic index and hepatosomatic index in the analysis and report the results. Gonadosomatic index (GSI; testes mass/somatic mass × 100%) was calculated using the nontransformed wet mass of testes and somatic tissue. Hepatosomatic index (HSI; liver mass/somatic mass × 100%) was calculated using the nontransformed dry mass of liver and dry mass of somatic tissue. Indices were first tested for normality, and while HSI failed this assumption, both indices were analyzed using generalized linear models with binomial error distributions (Warton & Hui, 2011), with environment and color morph as fixed effects and procedures as explained above. HSI had one extreme outlier (a fish with an extreme low value for liver dry mass from the estuarine environment). This data point was attributed to a data entry error and therefore excluded. We based this decision on the fact that the recorded wet mass of the same liver did not match the exceptionally low dry mass. Analyses were therefore done both with and without the outlier to control for the weight of the single datum point. Both analyses showed significantly lower HSI in the estuarine environment and the outlier was removed from the dataset to present a reliable unbiased sample.

Mean values in the text are given as back‐transformed values (if transformed for the statistical analysis) and presented with ± *SE*.

## 3 RESULTS

### 3.1 Effects of salinity and male origin on sperm velocity and viability

**FIGURE 1:**
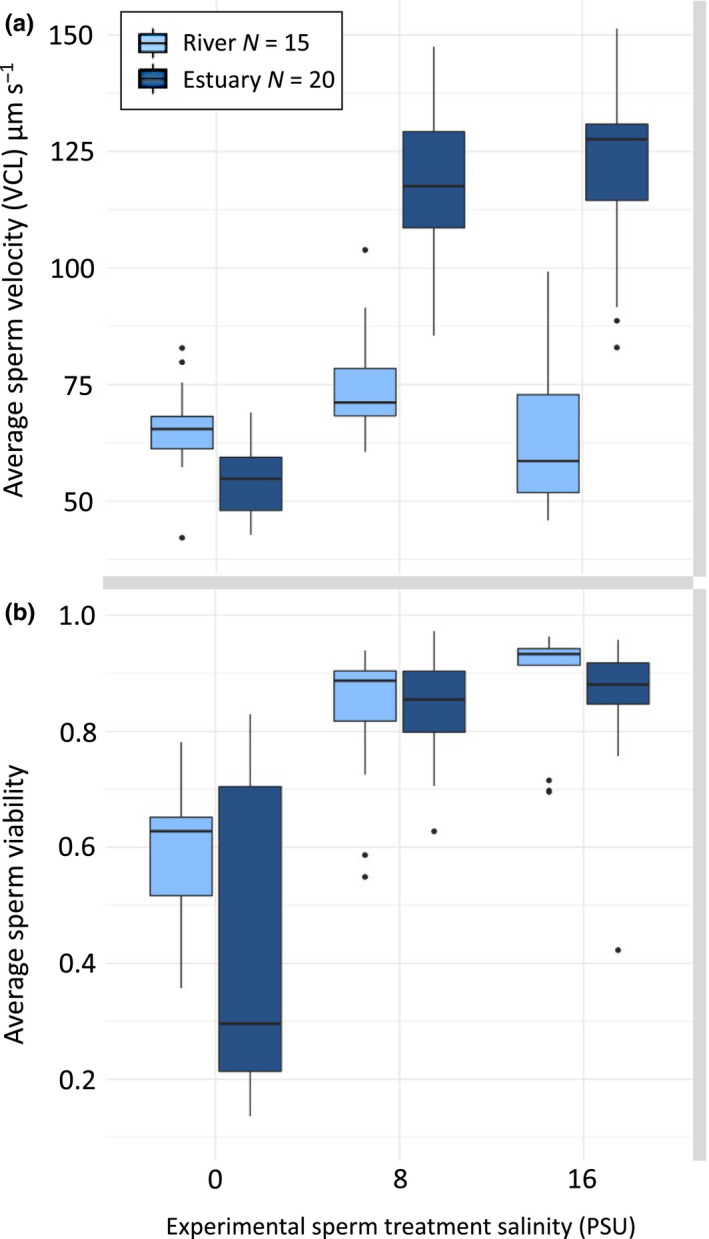
The effect of salinity on sperm velocity and viability. Sperm traits from round gobies (*N. melanostomus*) originating from river (0 PSU) (light blue, *N* = 15) and estuarine (~16 PSU) (dark blue, *N* = 20) environments. (a) Velocity and (b) viability of sperm, tested without sperm duct gland contents in three different salinities (0, 8, and 16 PSU). Boxplots show central line (median), and box range (first and third quantiles) together with whiskers (min and max values to a maximum of 1.5 times the distance between quantiles). Outliers are marked with small dots. Statistics are presented in Table 1a and b

In order to first test how sperm were affected by salinity, we analyzed the effects of salinity treatment (0, 8, and 16 PSU) and the environment that the males originated from (river or estuary) on sperm velocity. Salinity was found to strongly affect sperm velocity in interaction with male origin (Table 1a). We found higher velocity in higher salinities for estuarine males and relatively lower velocity for riverine males overall (Table 1a, Figure 1a). Furthermore, fish from both environments had comparatively low sperm velocity in freshwater (Figure 1a). Nevertheless, when tested in 0 PSU, sperm velocity was on average 12.1 µm/s higher for riverine (*N* = 15) than for estuarine fish (*N* = 20), but this difference was low compared to both 8 and 16 PSU, where sperm velocity was respectively 52.8 µm/s and 69.5 µm/s higher for estuarine males (Figure 1a). Hence, this shows that the sperm of riverine males handled fresh water (0 PSU) a little better than the sperm of estuarine males did, while the sperm of estuarine males handled brackish water (8 and 16 PSU) a lot better than the sperm of riverine males did.

**TABLE 1:** Linear mixed effects models analyzing effects of salinity on sperm performance in the round goby, *N. melanostomus*, from different environments

(a)								
Starting lme model: Sperm velocity ~ Salinity × Environment + (1 | Individual)								
Fixed effects	*S.S*.	*M.S*.	Num.*df*	Den.*df*	*F*	*p*	Sign.	Step
Salinity	27,898.0	13,949.0	2	66	98.345	<.0001	***	1
Environment	8,633.2	8,633.2	1	33	60.867	<.0001	***	1
Salinity × Environment	22,605.9	11,303.0	2	66	79.690	<.0001	***	1

Random effects	Variance	Std.Dev.						
Individual (35)	68.72	8.29						1
Error (Residual)	141.84	11.91						1
Number of obs: 105, groups: Individual, 35								

(b)								
Starting lme model: Sperm viability ~ Salinity × Environment + (1 | Individual)								
Fixed effects	*S.S*.	*M.S*.	Num.*df*	Den.*df*	*F*	*p*	Sign.	Step
Salinity	2.867	1.434	2	66	89.954	<.0001	***	1
Environment	0.039	0.039	1	33	2.465	.126	N.S.	1
Salinity × Environment	0.139	0.069	2	66	4.357	.017	*	1

Random effects	Variance	Std.Dev.						
Individual	0.008	0.086						1
Error (Residual)	0.060	0.126						1
Number of obs: 105, groups: Individual, 35								

Note

Tested response variables were (a) sperm velocity (VCL) and (b) sperm viability. Predictor variables were salinity treatment (3 levels: 1 PSU, 8 PSU, and 16 PSU) and environment (2 levels: riverine or estuarine) as fixed factors. Male ID was included as random factor to account for sperm from a single male being tested in several salinities. An × between variables denotes interactions. *p*‐values were generated in the lmerTest package from R using Satterthwaite approximations. Variables in italics show interactions that were removed during analysis (in order of highest *p*‐value, when nonsignificant). Error is based on the final model. *p*‐values are highlighted as follows: *p < .05, **p < .005, ***p < .001.

We also found that salinity treatment had a strong effect on sperm viability in interaction with male origin (Table 1b): when sperm were tested in fresh water, fish from the riverine environment showed significantly higher sperm viability than males from the estuarine environment (Tukey's range test, *p*_adj_ = 0.032) (Figure 1b). We also found a generally lower viability in 0 PSU compared to 8 and 16 PSU, for fish from both river and estuarine environments (Table 1b).

To also compare sperm performance between riverine and estuarine males in a salinity that corresponds to the local salinity they were caught in, and were likely to spawn in, we used the sperm velocity and viability data that were collected in 0 PSU for riverine males and 16 PSU for estuarine males. Sperm velocity was significantly lower for riverine males tested in 0 PSU (*N* = 15, 65.43 ± 2.49 µm/s) compared to males from the estuary tested in 16 PSU (*N* = 20, 121.28 ± 4.29 µm/s) (Welch *t* test, *t*_(29.5)_ = −11.249, *p* < .001). Similarly, when comparing sperm viability in their local salinity, sperm of riverine males tested in 0 PSU had overall lower viability (*N* = 15, 58.39 ± 3.27%) than sperm of estuarine males tested in 16 PSU (*N* = 20, 85.45 ± 2.60%) (Welch *t* test, *t*_(28.9)_ = −6.479, *p* < .001). This strongly suggests that the sperm of riverine males are less well adapted to their local salinity than the sperm of estuarine males.

### 3.2 Male color morphs

Comparing the relative occurrence of color morphs between the two environments (among the males that were assessed to be reproductively active and included in the experiment), we found that males in dark breeding color were equally common (chi‐square test, X^2^ = 3.04, *df* = 1, *p* = .08), although the nonsignificant trend was toward the dark morph being slightly more common among the riverine males (9 dark morph and 6 light morph males were caught in the river environment, and 5 dark and 15 light morph males in the estuarine environment). All dark males were estimated to be 3 years of age, but we also found light males that were 3 years old (river *N* = 2, estuary *N* = 9). All other males were 2 years old. Age estimate, total length, and body mass were analyzed for their effect on male color status. Body mass alone showed a significant effect in predicting color status, with heavier fish showing an increased likelihood of dark color (binary logistic regression model, X^2^ = 8.35, *df* = 1, *p* = .004). This indicates that male body mass determines color morph, likely because body mass determines nest‐holding ability and dark color reflects nesting behavior in this species.

### 3.3 The trade‐off between sperm velocity and sperm viability

**FIGURE 2:**
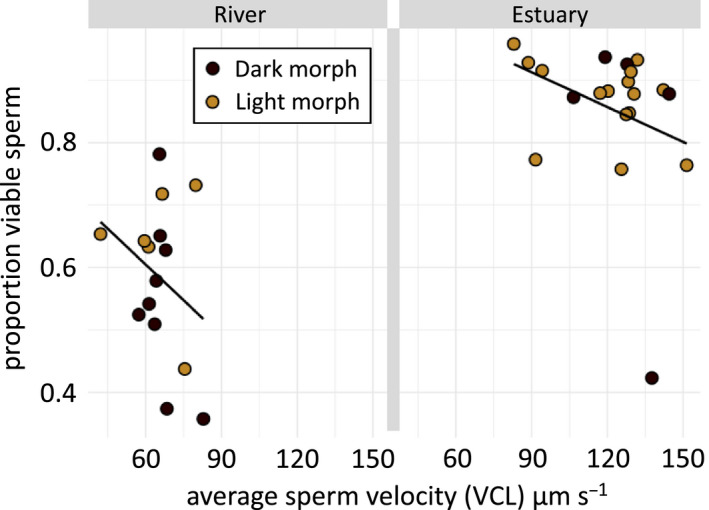
A trade‐off between sperm velocity and sperm viability. Sperm velocity of round goby (*N. melanostomus*) and its effects on sperm viability were tested in males from a freshwater river (left panel) and a brackish estuary (right panel) in light morph males (brown dots) and dark morph males (black dots). Sperm were tested in the salinity in which the males were caught (0 PSU for riverine and 16 PSU for estuarine). Regression lines in the figures were fitted with linear model in R: ggplot2. Statistics are presented in the results text

Since sperm viability can be expected to trade off with sperm velocity, we analyzed whether sperm velocity may affect sperm viability (assuming this being the more likely causal direction). We tested it using analysis of covariance, with sperm velocity as covariate. Color morph and environment were used as independent factors and sperm viability as the response variable. Full statistics can be found in Table 2. We found that increased sperm velocity reduced sperm viability (linear model, sperm velocity [covariate]: *F*_(1,31)_ = 26.71, *p* < .0001) (Figure 2), and that the males environment also had a significant effect (linear model, environment [factor]: *F*(1,31) = 23.47, *p* < .001), similar to the results presented in the previous section (Table 1b and Figure 1b). Viability did not differ between males of different color (linear model, color morph [factor]: *F*(1,31) = 2.29, *p* = .140) and color morph had no effect on the relationship of the trade‐off (linear model, sperm velocity [covariate] × color morph [factor]: *F*(1,31) = 0.062, *p* = .806). This shows that sperm trade off viability against velocity in both the freshwater river and the brackish estuary.

**TABLE 2:** Linear model showing the effects from sperm velocity on sperm viability in the round goby, *N. melanostomus*

Starting lm model: Sperm viability ~ Sperm velocity × Environment × Color morph						
Fixed effects	*S.S*.	Num.*df*	*F*	*p*	Sign.	Step
Sperm velocity	0.355	1	26.706	<.001	***	5
Environment	0.312	1	*23.470*	<.001	***	5
Color morph	0.031	1	2.293	.140	N.S.	5
*Sperm velocity* × *Environment*	0.004	1	0.272	.606	N.S.	4
*Environment* × *Color morph*	0.002	1	0.107	.746	N.S.	3
*Sperm velocity* × *Color morph*	0.001	1	0.062	.806	N.S.	2
*Sperm velocity* × *Environment* × *Color morph*	0.002	1	0.106	.747	N.S.	1
Error (Residual)	0.412	31				5

Note

The tested response variable was sperm viability. Predictor variables were color morph (dark or light), environment (riverine or estuarine) as factors, and sperm velocity (VCL) as covariate. An × between variables denotes interactions. Variables in italics show interactions that were removed during analysis (in order of highest *p*‐value, when nonsignificant). Error is based on the final model. *p*‐values are highlighted as follows: *p < .05, **p < .005, ***p < .001.

### 3.4 Effects of SDG contents and color morph on sperm velocity and viability

**FIGURE 3:**
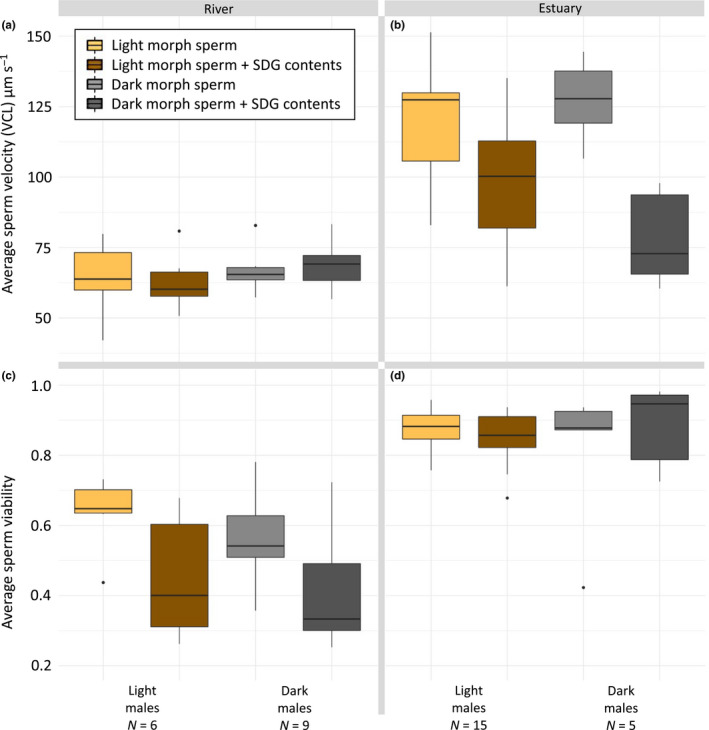
The effect of SDG contents treatment and male color morph on sperm velocity and viability. Sperm traits of round goby (*N. melanostomus*) were tested without and together with sperm duct gland contents in light morph males (brown colors) and dark morph males (gray colors). Sperm were tested in the salinity in which the males were caught (0 PSU for riverine and 16 PSU for estuarine). Panels show (a) sperm velocity of riverine males, (b) sperm velocity of estuarine males, (c) sperm viability of riverine males, and (d) sperm viability of estuarine males. Boxplot shows central line (median), and box range (first and third quantiles) together with whiskers (min and max values to a maximum of 1.5 times the distance between quantiles). Outliers are marked with small dots. Statistics are presented in Table 2a, b, c, and d

Since SDG contents may affect sperm and differ in their importance for different reproductive tactics, we analyzed the effects of SDG contents and color morph on sperm velocity and viability. Again, we focused on sperm that were tested in the local spawning salinity that the fish were caught in (0 PSU for riverine and 16 PSU for estuarine males), while analyzing the two groups separately. Full statistics are presented in Table 3. We found no effect from SDG and color morph on sperm velocity in riverine males (Table 3a, Figure 3a), but SDG had a strong negative effect in estuarine males, where dark males showed the strongest response (Table 3b, Figure 3b). SDG contents had a strong negative effect on sperm viability in riverine males, but there was no difference between color morphs (Table 3c, Figure 3c). For males from the estuarine environment, however, we found no effect from the SDG treatment on sperm viability at all (Figure 3d, Table 3d). In summary, for riverine males that spawn in fresh water, SDG contents decreased sperm viability. In contrast, for estuarine males that spawn in brackish water, SDG contents decreased sperm velocity.

**TABLE 3:** Linear mixed effects models analyzing effects of salinity on sperm performance in the round goby, *N. melanostomus* from different environments

(a)								
Starting lme model: VCL of riverine males ~ SDG Treatment × Colour morph + (1 | Individual)								
Fixed effects	*S.S*.	*M.S*.	Num.*df*	Den.*df*	*F*	*p*	Sign.	Step
SDG Treatment	2.693	2.693	1	14	0.0483	.8292	N.S.	2
Colour morph	43.691	43.691	1	13	0.7835	.3922	N.S.	2
*SDG Treatment* × *Colour morph*	16.417	16.417	1	13	0.279	.606	N.S.	1

Random effects	Variance	Std.Dev.						
*Individual*	35.63	5.969						2
Error (Residual)	55.77	7.468						2
Number of obs: 30, groups: Individual, 15								

(b)								
Starting lme model: VCL of estuarine ~ SDG Treatment × Colour morph + (1 | Individual)								
Fixed effects	*S.S*.	*M.S*.	Num.*df*	Den.*df*	*F*	*p*	Sign.	Step
SDG Treatment	8,797.1	8,797.1	1	18	76.698	<.001	***	1
Colour morph	58.7	58.7	1	18	0.512	.484	N.S.	1
SDG Treatment × Colour morph	1,641.1	1,641.1	1	18	14.308	.001	**	1

Random effects	Variance	Std.Dev.						
Individual	301.4	17.36						1
Error (Residual)	114.7	10.71						1
Number of obs: 40, groups: Individual, 20								

(c)								
Starting lme model: Viability of riverine males ~ SDG Treatment * Colour morph + (1 | Individual)								
Fixed effects	*S.S*.	*M.S*.	Num.*df*	Den.*df*	*F*	*p*	Sign.	Step
SDG Treatment	0.170	0.170	1	14	14.758	.002	**	2
Colour morph	0.008	0.008	1	13	0.652	.434	N.S.	2
*SDG Treatment* × *Colour morph*	0.007	0.007	1	13	0.552	.471	N.S.	1

Random effects:	Variance	Std.Dev.						
Individual	0.012	0.109						2
Error (Residual)	0.012	0.107						2
Number of obs: 30, groups: Individual, 15								

(d)								
Starting lme model: Viability of estuarine males ~ SDG Treatment * Colour morph + (1 | Individual)								
Fixed effects:	*S.S*.	*M.S*.	Num.*df*	Den.*df*	*F*	*p*	Sign.	Step
SDG Treatment	0.000	0.001	1	18.868	0.072	.792	N.S.	2
Colour morph	0.001	0.001	1	17.973	0.155	.699	N.S.	2
*SDG Treatment* × *Colour morph*	0.016	0.016	1	17.608	2.488	s.133	N.S.	1

Random effects:	Variance	Std.Dev.						
*Individual*	0.004	0.063						2
Error (Residual)	0.007	0.083						2
Number of obs: 39, groups: Individual, 20								

Note

Tested response variables were (a and b) sperm velocity (VCL) and (c and d) sperm viability. Males from the river (a and c) and estuary (b and d) were tested separately. Predictor variables were color morph (2 levels: dark or light), SDG treatment (2 levels: treatment with or without SDG contents) as fixed factors. Male ID was included as random factor to account for sperm from a single male being tested in several treatment conditions. An × between variables denotes interactions. *p*‐values were generated in the lmerTest package from R using Satterthwaite approximations. Variables in italics show interactions that were removed during analysis (in order of highest *p*‐value, when nonsignificant). Error is based on the final model. *p*‐values are highlighted as follows: *p < .05, **p < .005, ***p < .001.

### 3.5 Reproductive tissues

**FIGURE 4:**
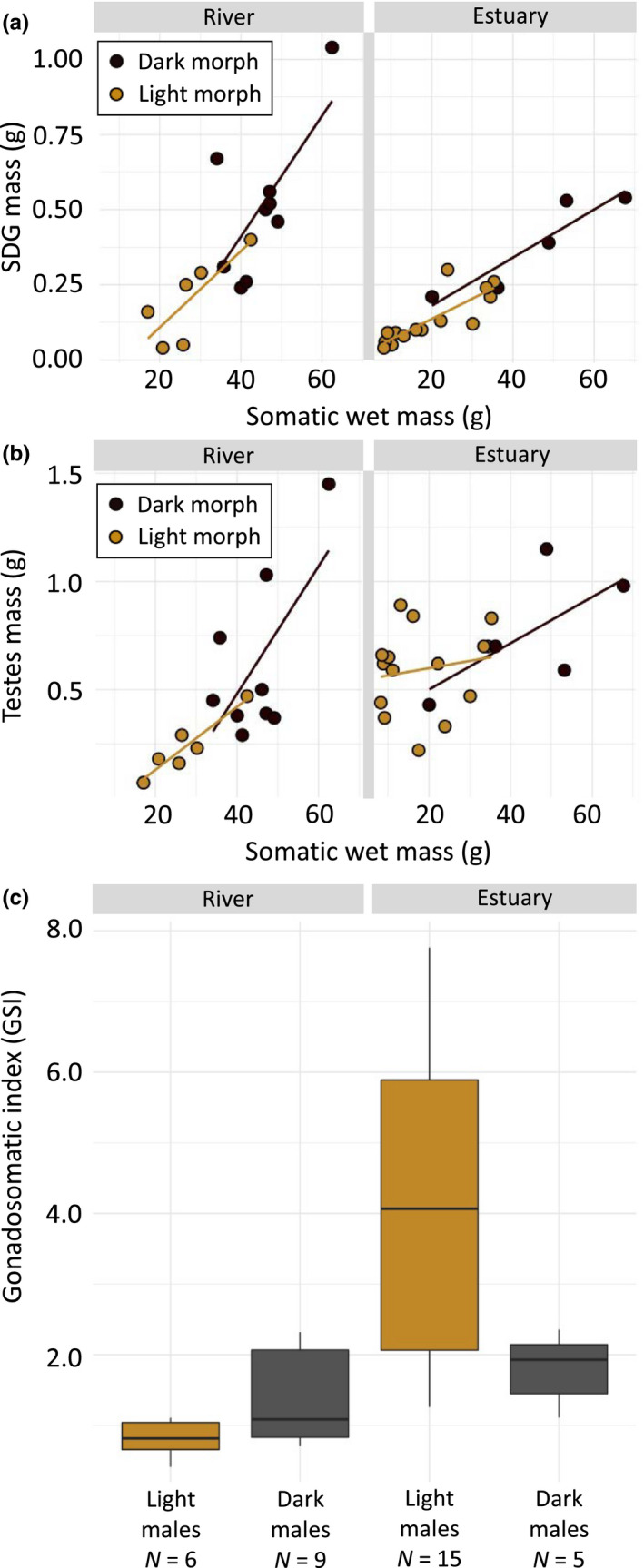
Reproductive tissues, (a) sperm duct gland (SDG) mass in grams and (b) testes mass in grams. Tissue masses covary with body size (somatic wet mass) for round goby (*N. melanostomus*) males of dark (black dots) and light (brown dots) color morphs from river (0 PSU) (dark *N* = 9, light *N* = 6) and estuarine (~16 PSU) (dark *N* = 5, light *N* = 15) environments. Regression lines in the figures were fitted with linear model in R: ggplot2. (c) Gonadosomatic index (GSI) is the testes mass divided by somatic body mass times 100. Boxplot shows central line (median), and box range (first and third quantiles) together with whiskers (min and max values to a maximum of 1.5 times the distance between quantiles). Statistics are presented in Table 3

Full statistics for factors affecting reproductive tissues are presented in Table 4. We found SDG mass to covary with somatic body mass and, given nonsignificant interaction terms, to scale similarly for both light and dark color morphs in both sampled salinity environments (Figure 4a). No significant effects of what environment the male originated from or color morph were found, but there was a nonsignificant trend (*p* = .055) toward heavier SDGs among dark males. Testes mass was also found to covary with somatic body mass, but differently so in the two different salinity environments, with a steeper slope among riverine than estuarine males (Figure 4b). Testes mass was overall higher for light males sampled in the estuarine environment (*N* = 15, 650.0 ± 51.2 µg) compared to the river (*N* = 6, 466.7 ± 93.5 µg). Expressed as GSI, there was a significant interaction between environment and color morph, such that light morph males from the estuary had higher GSI (*N* = 15) compared to the other male groups (Figure 4c). This suggests a high reproductive activity in light, presumably non‐nest‐holding, males in the estuary. In addition, environment affected GSI, with overall higher values for estuarine males, generated by a combination of higher testes mass and lower body mass (see below) in estuarine males.

**TABLE 4:** Linear models showing effects on reproductive tissues in the round goby, *N. melanostomus*

(a)						
Starting lme model: (log) SDG mass ~ Environment × Color morph × (log) Somatic wet mass						
Fixed effects	*S.S*.	*df*	*F*	*p*	Sign.	Step
Environment	0.000	1	0.004	.952	N.S.	5
Color morph	0.148	1	3.984	.055	N.S.	5
(log) Somatic wet mass (covariate)	1.256	1	33.689	<.001	***	5
*Environment* × *(log) Somatic wet mass*	0.107	1	3.073	.090	N.S.	4
*Color morph* × *(log) Somatic wet mass*	0.005	1	0.152	.700	N.S.	3
*Environment* × *Color morph*	0.000	1	0.000	.996	N.S.	2
*Environment* × *Color morph* × *(log) Somatic wet mass*	0.000	1	0.013	.911	N.S.	1
Error	1.042	27				5

(b)						
Starting lme model: (log) Testes mass ~ Environment × Color morph × (log) Somatic wet mass						
Fixed effects	*S.S*.	*df*	*F*	*p*	Sign.	Step
Environment	0.720	1	24.218	<.001	***	4
Color morph	0.007	1	0.234	.632	N.S.	4
(log) Somatic wet mass (covariate)	0.523	1	17.582	<.001	***	4
Environment × (log) Somatic wet mass	0.554	1	18.645	<.001	***	4
*Color morph* × *(log) Somatic wet mass*	0.023	1	0.764	.389	N.S.	3
*Environment* × *Color morph*	0.000	1	0.001	.976	N.S.	2
*Environment* × *Color morph* × *(log) Somatic wet mass*	0.027	1	0.856	.363	N.S.	1
Error	0.892	30				4

(c)						
Starting lme model: GSI ~ Environment × Color morph						
Fixed effects	*S.S*.	*df*	*F*	*p*	Sign.	Step
Environment	48.718	1	19.153	<.001	***	1
Color morph	6.032	1	2.372	.134	N.S.	1
Environment × Color morph	14.877	1	5.849	.022	*	1
Error	78.851	31				1

Note

Tested response variables were (a) wet mass of sperm duct glands (SDG) and (b) testes, and (c) gonadosomatic index (GSI). Predictor variables were color morph (dark or light), environment (riverine or estuarine) as factors, and male body size (somatic wet mass) as covariate in a and b. An × between variables denotes interactions. Variables in italics show interactions that were removed during analysis (in order of highest *p*‐value, when nonsignificant). Error is based on the final model. *p*‐values are highlighted as follows: *p < .05, **p < .005, ***p < .001.

### 3.6 Physiological status

Riverine males were larger than estuarine males, measured both as total length (river: 143.5 ± 4.4 mm; estuary: 123.5 ± 5.9 mm; general linear model, environment [factor]: *F*_(1, 32)_ = 6.374, *p* = .01) and total wet mass (river: *N* = 15, 42.5 ± 3.7 g; estuary: *N* = 20, 28.5 ± 4.2 g; general linear model, environment [factor]: *F*_(1, 32)_ = 7.532, *p* = .01). The energy reserves of riverine males were also higher, shown by the increased HSI compared to estuarine males (river: *N* = 15, 10.98 ± 2.20, estuary: *N* = 19, 5.28 ± 0.59; general linear model, environment [factor]: *F*_(1, 31)_ = 7.338, *p* = .011). Liver dry mass was also significantly different between environments when controlling for the effect of body mass (general linear model, environment [factor]: *F*_(1, 32)_ = 5.536, *p* = .026, somatic dry mass [covariate]: *F*_(1,32)_ = 13.31, *p* = .001). Given that age did not differ between the two male groups (river: *N* = 15, 2.73 ± 0.12 years, estuary: *N* = 20, 2.6 ± 0.11 years; general linear model, *F*_(1, 33)_ = 0.650, *p* = .426), this difference in size and energy reserves indicates that the sampled riverine fish had more energy available and also put more energy into somatic growth than the estuarine fish did.

## 4 DISCUSSION

We found that sperm velocity and viability decreased in fresh water, but contrary to our hypothesis (I) we found that SDG contents also further reduced viability for riverine males in fresh water, while it instead reduced sperm velocity for estuarine males in brackish water. Also contrary to our hypothesis (II), riverine males were not found to invest more in SDG mass. Fish from the river also invested more in somatic tissue and fat reserves, compared to fish from the estuary, resulting in larger males with higher energy reserves in the freshwater river than in the brackish estuary, which contrasts with our hypothesis (III). In the estuary, we also found that light‐colored males invested heavily in testes, evident by their high GSI.

SDG contents have been found to promote sperm velocity in other gobies (Green & Kvarnemo, 2019; Locatello et al., 2013; Poli et al., 2018), but we did not see this effect in our experiment. Previous studies have hypothesized that the reported positive effect by gland content on sperm velocity is an effect of added energy reserves in the ejaculate, or protection from adverse conditions (Green & Kvarnemo, 2019; Locatello et al., 2013; Poli et al., 2018). We did not find any positive effect from gland contents on sperm viability either. Instead, SDG contents negatively affected riverine males in their sperm viability and estuarine males in their sperm velocity (Figure 3). This result is interesting but hard to explain with our limited understanding of the mechanisms by which salinity (or lack thereof) affects sperm. One possible explanation could be that SDG contents have some positive effect on a trait that we omitted to measure (for example sperm velocity within the first seconds of ejaculation), and that this unknown benefit is traded off with the negative effect of SDGs we observed, dependent on the environment. With lower sperm velocity, are river gobies more reliant on sperm viability to enable fertilization? Further research is needed to explain these patterns. The link between sperm velocity and fertilization success is not experimentally established in the species (but there are indications of a positive correlation from other gobies; Svenssonet al., 2017). There is also a knowledge gap on whether or not our measured levels of sperm viability can limit fertilization success.

Our experiment did not replicate the viability essay over time, but studies of other species have shown that goby sperm can survive over 24 hr (Green & Kvarnemo, 2019), possibly as an adaptation to the prolonged female egg laying (several hours per female) reported in several goby species (Marconato et al., 1996). Whether or not high sperm viability over time also exists in round goby sperm remains to be tested. If it does, SDG contents could be of importance in keeping sperm alive long‐term, especially if the sperm are embedded in mucus at the onset of the long spawning event, as found in other species (e.g., Marconato et al., 1996). How long eggs remain fertilizable in round goby has to our knowledge not yet been tested, but studies of grass goby show some eggs to remain fertilizable for up to 40 hr (Scaggiante et al., 1999).

In gobies, SDG content can have other roles in reproduction than sperm performance, for example, it is also known to reduce bacterial growth (Giacomello, Marri, Marchini, Mazzoldi, & Rasotto, 2008). Presumably, this function helps to keep eggs laid in the nest healthy, and it explains why males continue to deposit mucus inside the nest, also after spawning. Consistent with this function, larger males, which are most likely to be nest holders, have larger SDG's (Immler et al., 2004; Kvarnemo et al., 2010; Rasotto & Mazzoldi, 2002; Scaggiante et al., 1999; Svensson & Kvarnemo, 2007). Hence, the observed increase in SDG mass with male somatic body mass found here (Figure 4a) was expected. The positive and linear relationship found between body mass and SDG mass in both color morphs also suggests an importance of maintaining SDG growth together with somatic growth. As a nesting opportunity can present itself on short notice (Meunier et al., 2009), investing continued resources into SDG growth is likely to pay off.

Males are expected to invest in the best combination of sperm velocity and viability, and these are commonly interpreted to be traded off against each other (Ball & Parker, 1996; Levitan, 2000; Møller, 1998; Stockley, Gage, Parker, & Møller, 1997). In concert with that, we found a negative relationship between sperm velocity and viability, indicating a trade‐off, arguably generated by reduced sperm viability resulting from increased sperm velocity. The trade‐off was found independent of color morph and in both environments (Figure 2). In the round goby living in the fresh water of the North American Great Lakes, sperm velocity has been found to decline faster for light than for dark males (Marentette et al., 2009). This difference could either be due to an initially high sperm velocity of light males that their experiment did not capture, or it could be an effect of investment in larger numbers of sperm (or possibly increased sperm viability, although not measured) rather than mechanisms that promote velocity. In other fish families, sperm from sneaker males have been shown to have higher ATP content and velocity, as in salmonids (Burness, Casselman, Schulte‐Hostedde, Moyes, & Montgomerie, 2004; Gage et al., 2004; Vladić & Järvi, 2001), lower velocity (Taborsky, Schütz, Goffinet, & Sander van Doorn, 2018), or higher longevity, as in sunfish (Neff, Fu, & Gross, 2003). Alternatively, they can be adapted to function better when in direct contact with substances from the nest holder's ejaculate (Locatello et al., 2013). Whether such an adaptation exists in the round goby is unknown, but based on our results showing negative effects of (their own) SDG contents on sperm velocity of both light and dark males in brackish water, and on sperm viability in fresh water, it appears unlikely.

**FIGURE 5:**
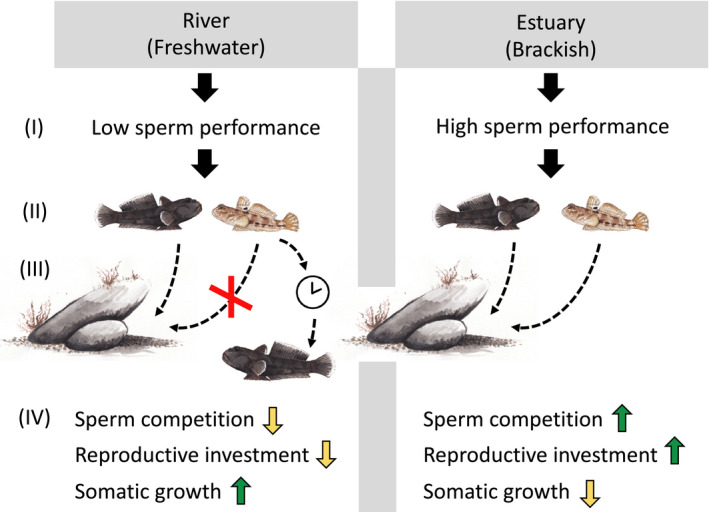
In the invasive round goby (*N. melanostomus*), we hypothesize that alternative reproductive tactics are associated with sperm performance in males from two different salinity environments. The figure presents a proposed scenario where low salinity causes cascade effects onto the expression of reproductive tactics. (I) Fresh water immediately lowers the velocity and viability of sperm when released into the water compared to in brackish conditions. (II) This limits the potential of fertilization success for sneaker tactics compared to nest‐holding tactics, where the male is closer to the unfertilized eggs and can control the timing of ejaculation better. (III) In the sampled freshwater environment, smaller males, which would otherwise employ parasitic spawnings through sneaking, instead invest in somatic tissue growth (size and mass) until they are large enough to hold a nest and court females themselves. The results are (IV) differences in sperm competition, differences in reproductive investment, and differences in somatic growth between males in the two environments.

Typically, males employing a sneaker tactic are confined to quick and imprecise fertilization attempts, with limited time to match sperm release to an egg depositing female (Taborsky, 2008). This promotes ejaculation of large numbers of sperm to swamp the nest‐holding males' sperm (Alonzo & Warner, 2000; Burness et al., 2004; Leach & Montgomerie, 2000; Neff et al., 2003; Pilastro & Bisazza, 1999; Scharer, 1999). When adverse environments compromise sperm velocity and viability, as in the freshwater conditions of river Elbe, we expect the sneaker tactic to be less successful. In such scenarios, sneaker behaviors are therefore likely to be selected against or never expressed in the behavioral and physiological repertoire, and therefore become rare in the population, compared to populations living in more benign environments (Dominey, 1984; Gross, 1991a; Gross & Repka, 1998). In the estuarine fish of our study, GSI was higher for light than for dark morph males (Figure 4c), offering a strong suggestion of sperm competition and expression of alternative reproductive tactics when sperm viability is high and parasitic fertilization attempts can pay off (Taborsky, 2008). Our average GSI value was 4.09 for estuarine light males, which is comparable to what was found previously for round goby sneaker males; a value of 4.22 (Marentette et al., 2009). Sperm competition has been proposed to be rife in fish since large sperm quantities are already promoted by external fertilization (Stockley et al., 1997; Taborsky, 2008). In comparison, sperm economy is favored during internal fertilization (Parker, 1984). In the hypothesized scenario described in Figure 5, we expect low sperm viability in fresh water to drive the spawning toward a situation more similar to internal reproduction, where a nest‐holding male is in control of the reproductive event, thus favouring sperm economy.

In gobies (including the round goby), the evidence supports plastically or ontogenetically induced tactics (Bleeker et al., 2017; Magnhagen, 1992; Somerville et al., 2019; Takegaki et al., 2012). The speed of plastic tactic change in round goby is unknown, and the current understanding of tactic changes and plasticity is so far based on observational data (Bleeker et al., 2017; Somerville et al., 2019) rather than experiments. Sneaker tactics in other goby species follow a continuum. In common gobies (*Pomatoschistus microps*, Krøyer 1838) the smallest males sneak, sometimes when also building their own nests, larger males build nests but do not sneak, and the largest males acquire their nests via nest take‐overs (Magnhagen, 1992). During our sampling, we only encountered dark males that were 3 years of age. This is an indication that a male will start out by reproducing through sneaking (when advantageous) and go on to adopt a nest‐holding strategy when large enough. The high GSI in a majority of the sampled estuarine light males supports that many of them reproduce via sneaking. However, there are also light‐colored males with higher somatic mass than dark males within the estuary. This suggests that some light‐colored males might be able to successfully overtake a nest, when the opportunity presents itself.

Similarly to what we found here, a previous study has shown the two male tactics to be size dependent (Bleeker et al., 2017). Bleeker et al. (2017) also reported a size‐related birthdate effect, arguing that a fish born late in the season would not have as much time to grow as their competitors that hatched earlier the same year. This has been further supported by less growth during the first year in round goby sneakers than in nest holders (McCallum et al., 2019). However, Somerville et al. (2019) analyzed round goby brain methylation patterns and found evidence of males starting to express a resource defending behavioral phenotype during favorable conditions (i.e., when they succeed in defending a potential nest), and not before this (i.e., not because of large size early in life).

Since sperm viability is low in the freshwater environment, we suggest that males are dependent on acquiring a nest site in order to reproduce successfully. Males are therefore expected to invest in somatic growth and energy reserves to be able to compete for and defend a nest site at a later time (Figures 5, IV). We found total length, wet mass, liver dry mass, and HSI to all be significantly higher in the river environment compared to the estuarine environment, supporting this expectation. Elsewhere, however, the round goby has been reported to be larger in brackish than in freshwater environments due to less energy being required for osmoregulation (Kornis et al., 2012; Sokołowska & Fey, 2011). We therefore originally predicted the river gobies to be of smaller size and in worse condition. Our current result is in contrast to this prediction and to previous studies, and it calls attention to complex dynamics between life‐history decisions related to growth and reproduction that likely are affecting the energy status of round goby in different salinities.

The low sperm velocity we report in 0 PSU is in contrast to a study from the Great Lakes (Marentette et al., 2009), where sperm velocity as high as in our 8 and 16 PSU treatments was observed in freshwater. In their study, and in the river Rhine (Bleeker et al., 2017), there is support for sneaker tactics being used. All light males that we sampled from the river habitat had less than 2% GSI, and according to Bleeker et al. (2017) therefore would not be classified as sneakers. The reports of sneaker morphs from freshwater in Marentette et al. (2009) and Bleeker et al. (2017) could reflect freshwater‐specific adaptations in the sperm of these populations, either ancestral (Vassilev, Apostolou, Velkov, Dobrev, & Zarev, 2012) or novel (Verliin et al., 2016). Indeed, round gobies in the Great Lakes, such as those studied by Marentette et al. (2009), have been found to be more closely related to freshwater populations than brackish populations (Brown & Stepien, 2009). If our river population is from a brackish ancestry, the patterns reported in our study reflect responses in a potentially mal‐adapted population. Our study also did not look at the reproductive traits of females. Ovarian fluid produced by females has been shown to help sperm function in a wide range of salinities in sticklebacks (Gasterosteidae) (Elofsson, Mcallister, Kime, Mayer, & Borg, 2003; Elofsson, Van Look, Sundell, Sundh, & Borg, 2006). Since ovarian fluid has shown to improve sperm velocity and life span of sperm in multiple families of fish (Zadmajid, Myers, Sørensen, & Ernest Butts, 2019), a role for it in goby reproductive ecology cannot be ruled out.

This study is limited first of all by the number of males sampled. Though considerable fishing effort was put into catching enough males to perform the study, the low numbers caught also highlight that reproductively active males can be comparatively few at any one site sampled. The study is also limited in its lack of replication across environments and can only point to differences between the two sites sampled. Other factors that may differ between these sites are unknown, in particular food availability and nest‐site availability. Food availability can drive growth rates, and in round goby, male nest‐holding tactics have been associated with time for growth in the first year between individuals of the same age cohort (McCallum et al., 2019; Somerville et al., 2019). Limited energy availability will also commonly limit reproductive investment. However, since higher energy reserves (in the form of HSI) were observed in the river than in the estuary, it is unlikely that riverine males were energy limited. Time since invasion is also an important factor for population density, and previous studies assessing population growth in round goby report densities to peak after 2–6 years of colonization and thereafter level off (Brownscombe & Fox, 2012). Since our populations were 9–11 years old, we expect both sampled sites to be at high but stable densities. During high population density, a lack of nest sites could drive a male to start employing sneaker tactics. The effect of nest availability on sneaking rates is yet to be experimentally tested in round goby. However, observational data suggest that environmental complexity (important for nest‐site availability) does not affect morph ratios (McCallum et al., 2019). In artificial aquarium conditions, round goby males of the light male morph have been reported to occupy a nest and court females when they did not have to compete with a dark, larger male (Meunier et al., 2009), suggesting that nest‐site availability might influence reproductive tactics. We did not provide sheltering that could be occupied in the holding tanks before sampling. This was to intentionally limit any light morph males from experiencing conditions (i.e., nest holding) that could trigger a switch in tactics. Though we estimate that the time in captivity did not affect morph ratios, we cannot be certain that the captive environment (lack of potential nest sites, lack of females, daily feeding) had no effect on the animals' physiology. Additionally, as we cut the testes and sperm duct glands to excise sperm and gland contents for the treatments, our sampling methods are artificial replicates of how the ejaculate would be mixed in vivo by the male. As a result, absolute values need to be interpreted with some caution. However, the error this method brings to the results is standardized across all our treatments, and sperm velocity and viability values are within the same ranges and comparable to previous studies, both using the method we describe above (Green et al., 2020; Green & Kvarnemo, 2019) and using sperm from stripped round goby (Marentette et al., 2009). An important benefit of our method is that it allowed us to test sperm without SDG contents present, which would have been hard to achieve with stripped sperm.

## 5 CONCLUSIONS

This study shows that local salinity conditions can decrease sperm velocity and viability for riverine gobies compared to estuarine gobies. The low sperm velocity and viability in fresh water likely limits male ability to spawn parasitically in such environments, since sneakers rely on sperm surviving and competing for fertilizations during the prolonged egg laying sequence of goby females. Consistent with this, none of the light‐colored round goby males from the freshwater environment had as high GSI as any of the light‐colored estuarine males had. Our results also show that although age did not differ between males from the two environments, the riverine males were larger and had higher energy reserves than the estuarine males, and together with the low GSI values, this suggests that riverine males invest their energy in growth rather than reproduction until they are large enough to become successful nest holders. In contrast, in the brackish salinity representing the estuarine environment, sperm velocity and viability were comparatively high, and light morph males with high GSI that are likely to be sneakers were common. These results thus suggest differences in life histories between the two environments regarding what reproductive tactic males employ. The ability of males to limit themselves from investing in sneak spawnings during unfavorable conditions and instead put energy into growth highlights a life‐history related trait that might promote the invasiveness of the species.

## CONFLICT OF INTEREST

The authors declare no competing interests.

## AUTHOR CONTRIBUTIONS

**Leon Green:** Conceptualization (lead); data curation (lead); formal analysis (lead); funding acquisition (supporting); investigation (lead); methodology (lead); project administration (lead); visualization (lead); writing—original draft (lead); writing‐review and editing (lead). **Jan Niemax**: Conceptualization (supporting); data curation (supporting); formal analysis (supporting); funding acquisition (supporting); methodology (supporting); writing—original draft (supporting); writing—review and editing (supporting). **Jens‐Peter Herrmann**: Conceptualization (supporting); funding acquisition (supporting); investigation (supporting); methodology (supporting); resources (supporting); writing—original draft (supporting); writing—review and editing (supporting). **Axel Temming**: Conceptualization (supporting); formal analysis (supporting); funding acquisition (lead); investigation (supporting); methodology (supporting); resources (lead); supervision (supporting); validation (supporting); writing—original draft (supporting); writing—review and editing (supporting). **Charlotta Kvarnemo**: Conceptualization (supporting); data curation (supporting); formal analysis (supporting); funding acquisition (lead); investigation (supporting); methodology (supporting); resources (lead); supervision (lead); validation (supporting); visualization (supporting); writing—original draft (supporting); writing—review and editing (supporting).

## ETHICAL APPROVAL

Experiments were conducted within the permit nr 59/16 from Amt für Vebraucherschutz, Veterinärwesen und Lebensmittelüberwachung, Hamburg.

## Data Availability

Raw data are available in the following repository: https://doi.org/10.5061/dryad.j0zpc86c0.
